# Association between Chinese herbal medicine (CHM) treatment and depression among cancer patients in China: An outpatient-based cross-sectional study

**DOI:** 10.1097/MD.0000000000034695

**Published:** 2023-08-25

**Authors:** Huiyue Lin, Xueting Zhang, Yi Zhang, Wenjing Cui, Fang Jia, Juyong Wang

**Affiliations:** a Oncology Department, Longhua Hospital Shanghai University of Traditional Chinese Medicine, Shanghai, China.

**Keywords:** cancer patients, Chinese herbal medicine, cross-sectional study, depression, health-related quality of life

## Abstract

Depression is a prevalent condition among cancer patients and significantly impacts their quality of life. Traditional Chinese Medicine, particularly Chinese Herbal Medicine (CHM), has shown potential in both anti-tumor and anti-depressive effects. However, there is a dearth of scientific literature exploring the association between CHM treatment and depression in cancer patients. This study aims to investigate the relationship between CHM treatment and depression in cancer patients. A cross-sectional study was conducted among cancer outpatients at Longhua Hosiptal, Shanghai University of Traditional Chinese Medicine, from June 2020 to April 2021 (Ethical approval number: 2020LCSY057). All patients signed informed consent and completed The European Organization for Research and Treatment of Cancer (EORTC) Quality of Life Questionnaire (EORTC QLQ-C30). Hamilton depression scale was evaluated depression by psychiatrists. The power of the sample size was determined using Gpower statistical and SPSS were used for statistical analysis. A total of 809 completed the study. Gender, medical insurance, the classification of time since diagnosis, ECOG, cancer stage, metastasis, gene mutation, treatment plan and CHM treatment were an important factor affecting depression (*P* < .05). Further analysis investigated the impact of CHM treatment on depression. There were 374 enrolled in CHM group and 435 enrolled in Non-CHM group. The assessment results of Hamilton depression scale and EORTC QLQ-C30 in CHM group were superior to those in Non-CHM group. The morbidity of depression is 50.27% in CHM group and 66.44% in Non-CHM group. After adjusting for potential confounders (gender, medical insurance, cancer stage, etc.), CHM treatment indicated negative correlation with depression (Odds ratio (OR) = 0.7, 95% confidence interval (CI): 0.5–0.9, *P* = .020). The interaction effects within each subgroup were no significantly affect the relationship between CHM treatment and depression (*P* > .05). CHM treatment was an independent protective factor for depression in cancer patients, and lead to better quality of life for cancer patients.

## 1. Introduction

According to the Global Cancer Observatory of the World Health Organization, there were approximately 19.29 million new cases of cancer and over 9.9 million cancer-related deaths worldwide in 2020.^[1]^ China alone accounted for around 4.5 million cancer cases and 3.0 million deaths, ranking it first in the world.^[1]^ Apart from physical, chemical, biological, and genetic factors, psychological factors, such as depression, play a role in the occurrence and development of malignant tumors. As early as 1957, a 20-year follow-up study on workers at the Western Power Company in the United States found that individuals who experienced emotional depression or instability were more likely to develop malignant tumors.^[2]^ Subsequent studies in 2000 demonstrated that negative emotions can impact the occurrence and progression of malignant tumors through psychological and physiological mechanisms.^[3]^ Therefore, it is evident that unhealthy mood can influence tumor development.

In modern society, with the rapid pace of development, unhealthy emotions, such as depression, have become increasingly prevalent. Depression is common among cancer patients, with prevalence rates ranging from 1.5% to 50%, which is 3 to 5 times higher than that observed in the general population.^[4]^ Cancer can increase susceptibility to depression through various avenues. The stressors of cancer diagnosis, complications, and the side effects of treatments like chemotherapy and radiotherapy can contribute to the development of depression. Conventional cancer treatments are often unable to eradicate the disease and cannot be used continuously, leading to feelings of abandonment and isolation among cancer patients who must discontinue or cannot tolerate these treatments. This, in turn, exacerbates symptoms such as depression.^[5]^ The coexistence of cancer and depression further deteriorates overall health. Current research has indicated that depression is an independent risk factor for cancer mortality, with estimates suggesting a 26% higher mortality rate among patients with depressive symptoms.^[6]^ Moreover, depression affects the quality of life, treatment compliance, and disease progression in cancer patients.^[7]^ Therefore, it is crucial to find ways to improve depression and enhance the quality of life, ultimately prolonging the longevity of cancer survivors.

Traditional Chinese Medicine (TCM) is a unique medical model in China and is widely recognized as an important complementary and alternative medicine with beneficial effects for cancer patients. Among TCM modalities, Chinese Herbal Medicine (CHM) plays a critical role in supporting cancer patients during their treatment.^[8,9]^ CHM is considered an alternative therapy for many cancer outpatients due to its effectiveness and minimal side effects.^[9]^ Importantly, CHM not only possesses anti-cancer properties but also exhibits anti-depressant effects. The anti-depressant and anti-cancer effects of CHM are mediated through various chemical components, multiple targets, and multiple pharmacological mechanisms.^[10,11]^ In contrast, the clinical evidence-based efficacy of antidepressant medications for treating depression in cancer patients and their effects on tumors are lacking, with relevant studies suggesting that antidepressant medication may lead to adverse side effects.^[11]^ Therefore, considering the aforementioned factors, CHM represents a favorable option for treating depression in cancer patients.

Currently, there is a lack of relevant research on the effects of CHM treatment on depression in cancer patients. Consequently, our study aims to preliminarily explore the relationship between CHM treatment and depression in cancer patients, with the goal of enhancing our understanding of depression in cancer patients and the influence of CHM on the depression.

## 2. Materials and Methods

### 2.1. Study population

**Figure 1.**
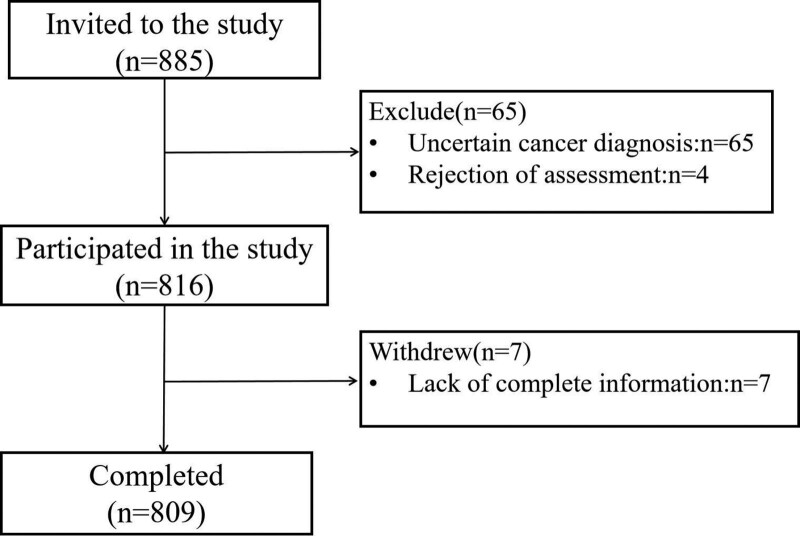
Flowchart of the study recruitment.

A total of 809 cancer outpatients were recruited and selected for this cross-sectional study conducted at Longhua Hospital, Shanghai University of Traditional Chinese Medicine, from June 2020 to April 2021 (Fig. 1). Exclusion criteria included: cancer diagnosis without cytological or pathological examination; loss of clinical data; poor communication ability; mental disorders caused by central nervous system disease; current use of psychotropic drugs; current hyperthyroidism or hypothyroidism. The study was conducted in accordance with the principles of the Declaration of Helsinki and approved by the Ethics Committee of Longhua Hospital, Shanghai University of Traditional Chinese Medicine, Shanghai, PRC (Approval Number: 2020LCSY057). All outpatients were provided with comprehensive information about the study objectives and procedures and provided informed consent.

### 2.2. Exposure of Chinese Herbal Medicine (CHM)

Certified Chinese medicine physicians provided all CHM treatments. The CHM group consisted of patients who received CHM treatment for more than 30 days, while the Non-CHM group did not receive CHM treatment. All cancer patients were administered the following basic formula: Astragalus mongholicus Bunge (Huang-qi), Largehead atractylodes rhizome (Bai-zhu), Tuckahoe (Fu-ling), Ternate pinellia rhizome (Ban-xia), and Ganoderma lucidum (Ling-zhi). Additional herbs were added to the basic formula based on the TCM practitioner’s diagnosis, considering the indications specific to each cancer patient.

### 2.3. Hamilton depression scale (HAMD)

The depression status of all outpatients was assessed using the HAMD-24,^[12]^ which is widely employed in Chinese clinical practice for evaluating depression. The HAMD-24 comprises 7 items related to anxiety/somatization, weight loss, diurnal variation, cognitive disturbance, retardation, sleep disturbance, and depression. Two trained psychiatrists conducted face-to-face interviews to evaluate the HAMD-24 scores. Patients with HAMD scores above 8 were considered to have depressive symptoms.

### 2.4. Health-related quality of life (HRQoL)

The European Organization for Research and Treatment of Cancer (EORTC) Core Quality of Life Questionnaire (QLQ-C30) was utilized in this study.^[13]^ The Chinese version of the EORTC QLQ-C30 (version 3.0) has been validated as a reliable instrument for assessing the quality of life of Chinese cancer patients.^[14]^ The questionnaire includes functional scales such as physical, role, emotional, cognitive, and social functioning, as well as symptom sub-scales such as pain, fatigue, nausea and vomiting, and global health status. Individual measurement items include appetite, insomnia, dyspnea, constipation or diarrhea, and economic status. The HRQoL was assessed using the EORTC QLQ-C30 (Chinese version 3.0) questionnaire, which was completed by the outpatients themselves. Illiterate patients or those with writing difficulties could receive assistance from doctors in completing the questionnaire. The standardized HRQoL scores were calculated on a scale of 0 to 100, where higher scores indicated better functional level, higher global quality of life, and more severe symptoms.^[15]^

### 2.5. Statistical analysis

Variables following a normal distribution were described as mean ± SD and analyzed using One-Way ANOVA to determine statistical differences. Skewed distribution variables were presented as median (quartile) and analyzed using the Mann–Whitney *U* test. Qualitative variables were described as frequency or percentage and analyzed using chi-square tests. Binary logistic regression models were used to evaluate the associations between CHM treatment and depression status, with both non-adjusted and multivariate adjusted models presented. Covariates were included in the model if their addition changed the matched odds ratio by at least 10%, following the recommendations of the STROBE statement.^[16]^ Subgroup analyses were conducted using stratified linear regression models, with the likelihood ratio test used to examine modifications and interactions within subgroups. Sample size calculations were performed using G*Power 3.1.9.7 statistical power analysis software (https://www.gpower.hhu.de/en.html). The type 1 and type 2 errors were set at 0.05 and 80%, respectively, and the effect size was set at 0.15. A *P* value of < .05 was considered statistically significant.^[17]^ Considering 10 factors, the power analysis indicated that a total sample size of 705 subjects would be required to detect a significant difference. All analyses were conducted using SPSS version 26.0 (Chicago, USA). *P* values less than .05 (two-sided) were considered statistically significant.

## 3. Results

### 3.1. Baseline characteristics of participants

A total of 809 cancer outpatients were recruited for this cross-sectional study conducted between June 2020 and April 2021 (Table 1). Among them, 477 cancer patients exhibited symptoms of depression, while 332 cancer patients did not.

**Table 1:** Baseline characteristics of participants (N = 809).

Characteristics	CHM
Age (yr)	
<60	458 (56.61)
≥60	351 (43.39)
Medical insurance	
Self-financed patient	346 (42.77)
Medicare patient	463 (57.23)
Gender	
Female	556 (68.73)
Male	253 (31.27)
ECOG PS	
<1	447 (55.25)
≥1	362 (44.75)
Cancer stage*	
I	407 (50.31)
II	176 (21.75)
III	108 (13.35)
IV	118 (14.59)
Cancer type	
Lung Cancer	400 (49.44)
Breast Cancer	190 (23.49)
Thyroid Cancer	97 (11.99)
Digestive system tumors†	75 (9.27)
Other types of cancer‡	47 (5.81)
Metastasis at diagnosis	
No	479 (59.21)
Yes	330 (40.79)
Gene mutation	
Untested	674 (83.31)
Yes	135 (16.69)
The classification of time since diagnosis (months)	
T ≤ 12	411 (50.80)
T > 12	398 (49.20)
Treatment plan at visit	
Only TCM	253 (31.27)
TCM with adjuvant therapy§	30 (3.71)
TCM with endocrine therapy‖	91 (11.25)
Only adjuvant therapy§	109 (13.47)
Only endocrine therapy‖	85 (10.51)
No therapy	241 (29.79)
State of depression	
No	332 (41.04)
Yes	477 (58.96)

CHM = Chinese herbal medicine, ECOG PS = Eastern Cooperative Oncology Group performance status, HAMD = Hamilton depression scale, TCM = Traditional Chinese Medicine.

* Clinical stage classified on the basis of on the American Joint Committee on Cancer 7th edition staging system.

† Digestive system tumors included gastric carcinoma, colorectal carcinoma, hepatocarcinoma, pancreatic carcinoma, gallbladder carcinoma, esophageal carcinoma.

‡ Other types of cancer included ovarian carcinoma, cervical carcinoma, bladder carcinoma, non-Hodgkin’s lymphoma.

§ Adjuvant treatment included chemotherapy, radiotherapy, targeted therapy, immunotherapy.

‖ Endocrine treatment included estrogen therapy, thyroid stimulating hormone suppression therapy.

### 3.2. Univariate analysis of depression

The results of the univariate analysis revealed that gender, medical insurance, time since diagnosis, performance status of Eastern Cooperative Oncology Group (ECOG PS), cancer stage (III, IV), metastasis at diagnosis, gene mutation, adjuvant treatment, and CHM treatment showed correlations with depression (*P* < .05). However, age and cancer type did not exhibit significant associations with depression (*P* > .05) (Table 2).

**Table 2:** Univariate analysis for depression.

Covariate	Statistics	OR (95% CI)	*P* value
Age (years)			
<60	458 (56.61)	Reference	
≥60	351 (43.39)	0.9 (0.7, 1.2)	.88
Gender			
Female	556 (68.7%)	Reference	
Male	253 (31.3%)	0.6 (0.5, 0.9)	**<.01**
Medical insurance			
Self-financed patient	346 (42.8%)	Reference	
Medicare patient	463 (57.2%)	0.7 (0.5, 0.9)	**<.01**
The classification of time since diagnosis (mo)			
T ≤ 12	411 (50.80)	Reference	
T > 12	398 (49.20)	0.7 (0.5, 0.9)	**<.05**
ECOG PS			
<1	447 (55.3%)	Reference	
≥1	362 (44.7%)	1.6 (1.2, 2.1)	**<.01**
Cancer stage			
I	407 (50.3%)	Reference	
II	176 (21.8%)	1.3 (0.9, 1.8)	.17
III	108 (13.3%)	1.6 (1.0, 2.4)	**<.05**
IV	118 (14.6%)	1.9 (1.2, 2.9)	**<.01**
Cancer type			
Lung cancer	400 (49.4%)	Reference	
Breast cancer	190 (23.5%)	1.1 (0.8, 1.5)	.68
Thyroid cancer	97 (12.0%)	0.9 (0.6, 1.4)	.58
Digestive system tumors	75 (9.3%)	0.9 (0.6, 1.6)	.82
Other cancer	47 (5.8%)	1.2 (0.7, 2.3)	.50
Metastasis at diagnosis			
No	479 (59.2%)	Reference	
Yes	330 (40.8%)	1.6 (1.2, 2.2)	**<.01**
Gene mutation			
Untested	674 (83.3%)	Reference	
Yes	135 (16.7%)	1.8 (1.2, 2.7)	**<.01**
Treatment plan at visit			
Without other treatment	494 (61.1%)	Reference	
Adjuvant treatment	139 (17.2%)	1.8 (1.2, 2.6)	**.01**
Endocrine treatment	176 (21.8%)	1.2 (0.8, 1.7)	.38
Treatment of CHM			
No	435 (53.77%)	Reference	**<.01**
Yes	374 (46.23%)	0.5 (0.4, 0.7)	

Nominally significant *P* values (*P* < .05) are denoted in bold.

CI = confidence interval, CHM = Chinese herbal medicine, ECOG PS = Eastern Cooperative Oncology Group performance status, OR = odds ratio.

### 3.3. Characteristics of participants in CHM group and Non-CHM group

**Figure 2.**
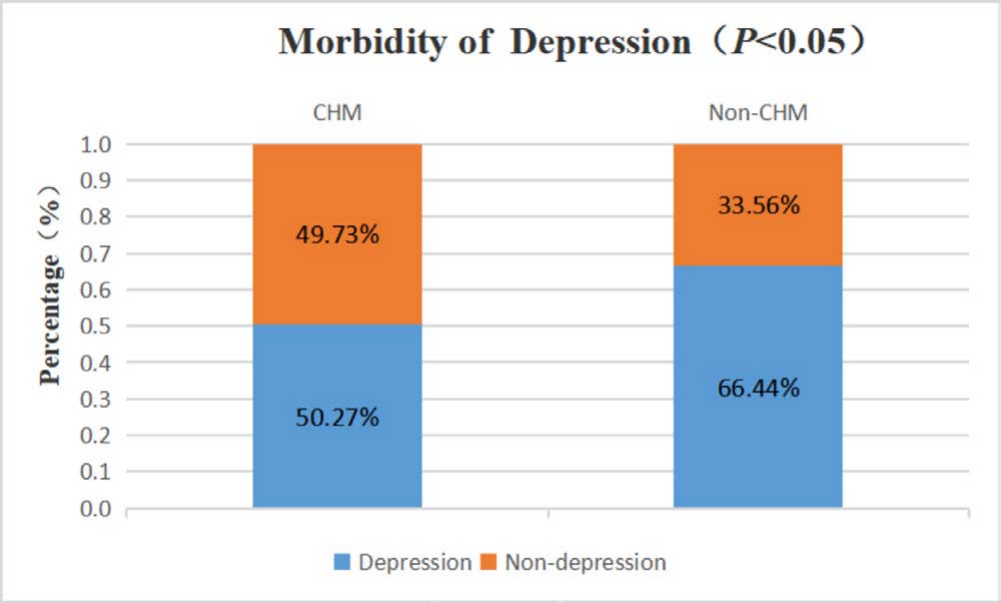
The morbidity of depression in CHM group and Non-CHM group. CHM = Chinese herbal medicine.

A total of 374 participants were enrolled in the CHM group, while 435 participants were enrolled in the Non-CHM group. There was no statistically significant difference in gender distribution between the 2 groups. However, significant differences were observed in the distribution of age, medical insurance, ECOG PS, clinical stage, cancer type, metastasis, gene mutation, and the classification of time since diagnosis and treatment plan between the 2 groups (Table 3). The ratio of depression status in the CHM group and Non-CHM group was 50.27% and 66.44%, respectively (Fig. 2, *P* < .05).

**Table 3:** Characteristics of Participants in CHM group and Non-CHM group.

Characteristics	CHM	Non-CHM	*P* value
Number	374	435	
Age, n (%)			
<60	194 (51.87)	264 (60.69)	**.012**
≥60	180 (48.13)	171 (39.31)	
Medical insurance, n (%)			
Self-financed patient	117 (31.28)	229 (52.64)	**<.001**
Medicare patient	257 (68.72)	206 (47.36)	
Gender, n (%)			
Female	253 (67.65)	303 (69.66)	.539
Male	121 (32.35)	132 (30.34)	
ECOG PS, n (%)			
<1	245 (65.51)	202 (46.44)	**<.001**
≥1	129 (34.49)	233 (53.56)	
Clinical stage*, n (%)			
I	235 (62.83)	172 (39.54)	**<.001**
II	81 (21.66)	95 (21.84)	
III	30 (8.02)	78 (17.93)	
IV	28 (7.49)	90 (20.69)	
Cancer type, n (%)			
Lung cancer	174 (46.52)	226 (51.95)	**.040**
Breast cancer	98 (26.20)	92 (21.15)	
Thyroid cancer	54 (14.44)	43 (9.89)	
Digestive system tumors†	32 (8.56)	43 (9.89)	
Other types of cancer‡	16 (4.28)	31 (7.13)	
Metastasis at diagnosis, n (%)			
No	270 (72.19)	209 (48.05)	**<.001**
Yes	104 (27.81)	226 (51.95)	
Gene mutation, n (%)			
Untested	341 (91.18)	333 (76.55)	**<.001**
Yes	33 (8.82)	102 (23.45)	
The classification of time since diagnosis (mo), n (%)			
T ≤ 12	86 (22.99)	325 (74.71)	**<.001**
T > 12	288 (77.01)	110 (25.29)	
Treatment plan at visit, n (%)			
Without other treatment	253 (67.65)	241 (55.40)	**<.001**
Adjuvant treatment§	30 (8.02)	109 (25.06)	
Endocrine treatment‖	91 (24.33)	85 (19.54)	

Nominally significant *P* values (*P* < .05) are denoted in bold.

CHM = Chinese herbal medicine, ECOG PS = Eastern Cooperative Oncology Group performance status, HAMD = Hamilton depression scale.

* Clinical stage classified on the basis of on the American Joint Committee on Cancer 7th edition staging system.

† Digestive system tumors included gastric carcinoma, colorectal carcinoma, hepatocarcinoma, pancreatic carcinoma, gallbladder carcinoma, esophageal carcinoma.

‡ Other types of cancer included ovarian carcinoma, cervical carcinoma, bladder carcinoma, non-Hodgkin’s lymphoma.

§ Adjuvant treatment included chemotherapy, radiotherapy, targeted therapy, immunotherapy.

‖ Endocrine treatment included estrogen therapy, thyroid stimulating hormone suppression therapy.

### 3.4. The results of HAMD scores and HRQoL

Compared to the Non-CHM group, HAMD scores were significantly decreased In the CHM group (*P* < .001). Additionally, the CHM group showed significantly higher mean scores in physical functioning, role functioning, emotional functioning, social functioning, and global health status compared to the Non-CHM group (*P* < .05). Conversely, the Non-CHM group exhibited significantly higher mean scores in symptoms such as fatigue, nausea and vomiting, pain, dyspnea, insomnia, appetite loss, constipation, and financial difficulties compared to the CHM group (*P* < .05). No significant differences were observed in cognitive functioning (*P* = .079) and diarrhea (*P* = .190) between the 2 groups (Table 4).

**Table 4:** The results of HAMD scores and HRQoL.

Outcomes	CHM			Non-CHM			*P* value
	N	Mean (SD)	Median (Q1–Q3)	N	Mean (SD)	Median (Q1–Q3)	
HAMD scores	374	9.9 (5.2)	9.0 (5.0–12.0)	435	11.7 (5.9)	11.0 (8.0–15.0)	**<.001**
EORTC QLQ-C30							
Physical functioning	374	84.0 (13.2)	86.7 (80.0–93.3)	435	81.6 (15.3)	86.7 (73.3–93.3)	**.040**
Role functioning	374	91.3 (15.5)	100.0 (83.3–100.0)	435	81.9 (22.0)	100.0 (66.7–100.0)	**<.001**
Emotional functioning	374	85.5 (14.4)	91.7 (81.2–100.0)	435	78.2 (17.0)	83.3 (66.7–91.7)	**<.001**
Cognitive functioning	374	82.1 (16.0)	83.3 (66.7–100.0)	435	80.0 (17.1)	83.3 (66.7–100.0)	.079
Social functioning	374	90.2 (16.0)	100.0 (66.7–100.0)	435	81.5 (21.2)	100.0 (66.7–100.0)	**<.001**
Global health status	374	70.3 (12.5)	66.7 (66.7–83.3)	435	67.5 (14.2)	66.7 (66.7–83.3)	**.003**
Fatigue	374	23.2 (20.9)	22.2 (0.0–33.3)	435	31.9 (20.8)	33.3 (11.1–44.4)	**<.001**
Nausea and vomiting	374	3.7 (13.3)	0.0 (0.0–0.0)	435	4.7 (12.8)	0.0 (0.0–0.0)	**.026**
Pain	374	12.0 (20.3)	0.0 (0.0–16.7)	435	15.4 (17.5)	16.7 (0.0–33.3)	**<.001**
Dyspnea	374	11.3 (19.2)	0.0 (0.0–33.3)	435	16.0 (21.1)	0.0 (0.0–33.3)	**<.001**
Insomnia	374	24.8 (27.6)	33.3 (0.0–33.3)	435	31.6 (29.6)	33.3 (0.0–66.7)	**.001**
Appetite loss	374	6.7 (17.1)	0.0 (0.0–0.0)	435	13.0 (21.8)	0.0 (0.0–33.3)	**<.001**
Constipation	374	6.1 (16.3)	0.0 (0.0–0.0)	435	10.7 (19.8)	0.0 (0.0–33.3)	**<.001**
Diarrhea	374	8.0 (18.0)	0.0 (0.0–0.0)	435	6.6 (16.4)	0.0 (0.0–0.0)	.190
Financial difficulties	374	9.8 (17.2)	0.0 (0.0–33.3)	435	13.9 (19.7)	0.0 (0.0–33.3)	**<.001**

Nominally significant *P* values (*P* < .05) are denoted in bold.

CHM = Chinese herbal medicine, EORTC QLQ-C30 = The European Organization for Research and Treatment of Cancer (EORTC) Core Quality of Life Questionnaire (QLQ-C30), HAMD = Hamilton depression scale.

### 3.5. The results of relationship between CHM treatment and depression

The associations between CHM treatment and depression were evaluated using a binary logistic regression model. In the crude model, receiving CHM treatment was negatively correlated with depression (OR = 0.5, 95% CI: 0.4–0.7, *P* < .001). After adjusting for ECOG PS, cancer stage, gene mutation, metastasis at diagnosis, time since diagnosis, and treatment plan at visit (Model I), the result did not change significantly (OR = 0.6, 95% CI: 0.4–0.8, *P* = .003). Moreover, even in Model II, which further incorporated factors like gender and medical insurance, the aforementioned trends persisted (OR = 0.7, 95% CI: 0.5–0.9, *P* = .020). In sensitivity analysis, when the duration of CHM treatment was classified and handled as categorical variables, a similar trend was observed (*P* for trend: <0.01, 0.028, 0.128 in crude model, Model I, Model II, respectively) (Table 5).

**Table 5:** Relationship between CHM treatment with depression.

Variable	Crude model		Model I		Model II	
	OR (95% CI)	*P* value	OR (95% CI)	*P* value	OR (95% CI)	*P* value
CHM treatment						
Non-received	Reference		Reference		Reference	
Received	0.5 (0.4, 0.7)	**<.001**	0.6 (0.4, 0.8)	**.003**	0.7 (0.5, 0.9)	**.020**
Duration of CHM treatment (mo)						
0	Reference		Reference		Reference	
≤12	0.6 (0.4, 0.8)	**.001**	0.6 (0.4, 0.9)	**.012**	0.7 (0.5, 1.0)	**.047**
12–24	0.5 (0.3, 0.7)	**.001**	0.5 (0.3, 0.9)	**.024**	0.6 (0.3, 1.0)	.058
>24	0.5 (0.3, 0.7)	**.001**	0. 6 (0.3, 0.9)	**.029**	0.6 (0.4, 1.1)	.113
*P* for trend		**<.001**		**.028**		.128

Nominally significant *P* values (*P* < .05) are denoted in bold.

CHM = Chinese herbal medicine, CI = confidence interval, ECOG PS = Eastern Cooperative Oncology Group performance status, OR = odds ratio.

Model I adjusted for ECOG PS, cancer stage, gene mutation, metastasis at diagnosis, time since diagnosis, treatment plan at visit.

Mode II adjusted for gender, medical insurance, ECOG PS, cancer stage, gene mutation, metastasis at diagnosis, time since diagnosis, treatment plan at visit.

### 3.6. The results of subgroup analyses

**Figure 3.**
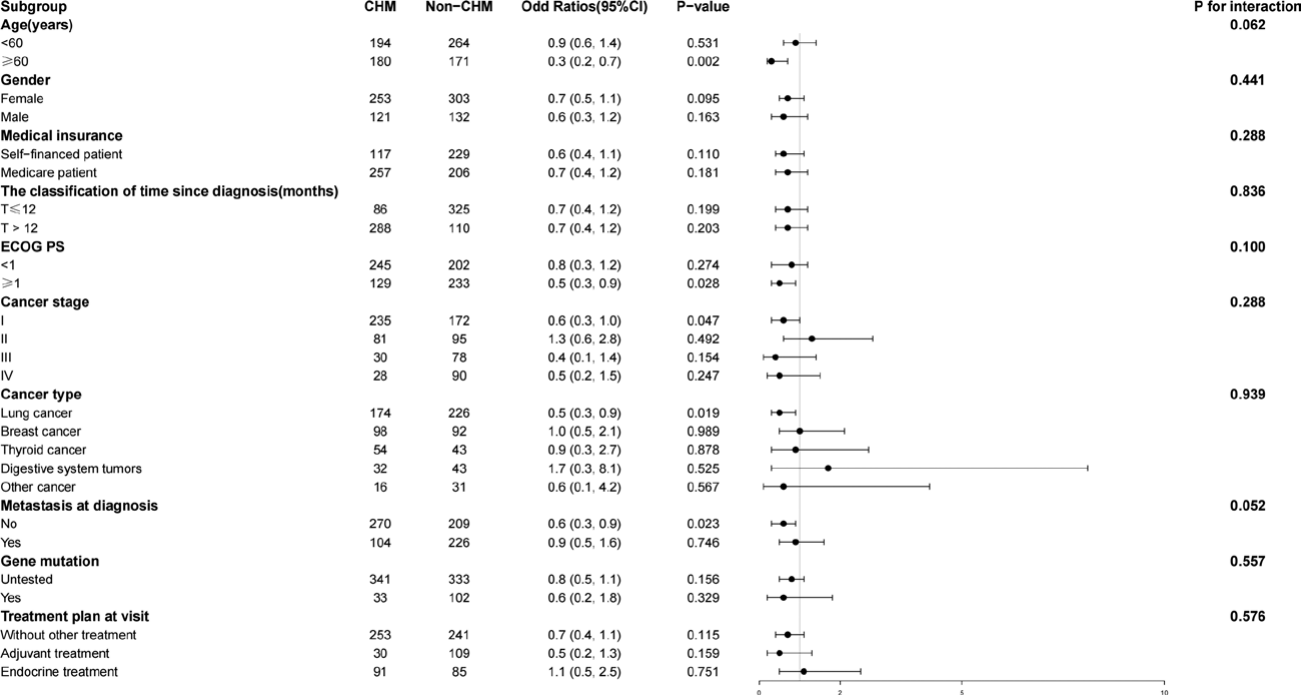
Effect size of CHM treatment on depression in prespecified and exploratory subgroups in each subgroup. CHM = Chinese herbal medicine, ECOG PS = performance status of Eastern Cooperative Oncology Group.

The potential interactions between CHM treatment and various subgroup variables were examined. The test for interactions in each subgroup, including metastasis at diagnosis, age, gender, medical insurance, time since diagnosis, ECOG PS, cancer stage, gene mutation, and treatment plan at visit, did not show statistically significant effects on the relationship between CHM treatment and depression after adjusting for relevant confounders, except the subgroup variable (*P* for interaction = 0.062, 0.441, 0.288, 0.836, 0.100, 0.288, 0.939, 0.052, 0.557 and 0.576, respectively) (Fig. 3).

## 4. Discussion

The objective of this study was to investigate the association between CHM treatment and depression in cancer outpatients. Our assessment of HAMD scores and EORTC QLQ-C30 outcomes revealed better results in the CHM group compared to the Non-CHM group (*P* < .05). After adjusting for confounding factors in Model II, CHM treatment showed a significant negative association with depression (OR = 0.7, 95% CI: 0.5–0.9, *P* = .020). Sensitivity analysis supported the consistent trend. Furthermore, the interactions in each subgroup did not significantly affect the relationship between CHM treatment and depression (*P* > .05). These findings suggest that CHM treatment can effectively alleviate depression in cancer patients, thereby improving their quality of life. Overall, combined with the results of our hierarchical analysis, CHM treatment emerged as an independent protective factor against depression in cancer patients.

A total of 809 cancer outpatients were enrolled in this cross-sectional study, comprising 477 patients with depression and 332 patients without depression. Univariate analysis identified several factors associated with the depression status in cancer patients. Specifically, gender, medical insurance, time since cancer diagnosis, ECOG PS, cancer stage (III, IV), metastasis at diagnosis, gene mutation, adjuvant treatment, and CHM treatment exhibited correlations with depression status. While many of these findings are consistent with previous studies, some discrepancies may arise from differences in race, medical environment, and understanding of tumors.^[18–21]^ Notably, a study has demonstrated the effectiveness of CHM treatment in reducing the risk of depression in cancer patients.^[22]^ Our study also yielded similar results, further supporting the potential benefits of CHM treatment in alleviating depression among cancer patients.

We conducted a study with a total of 809 patients, dividing them into the CHM group and the Non-CHM group, aiming to investigate the association between CHM and depression. The results indicated that there was no significant difference between the 2 groups in only terms of gender, suggesting that these factors may influence the relationship between CHM and depression. To further elucidate the impact of CHM treatment on depression in cancer patients, we performed binary logistic regression analysis. After adjusting for potential confounders such as ECOG PS, cancer stage, gene mutation, metastasis at diagnosis, time since diagnosis, and treatment plan at visit, the odds ratio (OR) for CHM treatment was less than 1 (*P* < .05), and the OR for the duration of CHM treatment gradually decreased, with a significant trend (*P* for trend < .05). Although the full adjustment of confounders (gender, medical insurance, ECOG PS, cancer stage, gene mutation, metastasis at diagnosis, time since diagnosis, treatment plan at visit) did not reach statistical significance (*P* > .05), the OR remained consistent, indicating a stable relationship between CHM treatment and depression status. The binary logistic regression model revealed that CHM treatment was associated with a lower risk of depression in cancer patients (OR = 0.7, 95% CI: 0.5–0.9, *P* = .020). The subgroup analyses were crucial for our scientific investigation, and the results indicated that none of the subgroups significantly influenced the relationship between CHM treatment and depression (*P* > .05), suggesting that the effect of CHM treatment on depression in cancer patients remains consistent across different subgroups. Therefore, CHM treatment can be considered an independent protective factor against depression in cancer patients.

In our study, the results demonstrated that HAMD scores in the CHM group were significantly decreased compared with Non-CHM group (*P* < .001). Although received CHM treatment could alleviate depression of cancer patients, depression still persisted. The presence of tumor recurrence and various adverse symptoms during the course of disease development and progression can contribute to depression in cancer patients.^[23]^ The Hamilton Depression Scale is commonly used for clinical depression diagnosis. However the recognized evaluation scale for depression in cancer patients had yet to be established. Nevertheless, the Hamilton Depression Scale remains widely used for diagnosing depression in cancer patients and provides a reliable assessment of their depressive status.^[24,25]^ The quality of life in cancer patients has always been the focus of attention of clinicians. Consequently, several HRQoL tools have been developed and validated for the cancer population, including the European Organization for the Research and Treatment of Cancer QLQ-C30 (EORTC QLQ-C30). Currently, EORTC QLQ-C30 is extensively used to measure quality of life in cancer patients in clinical trial.^[2]^ In our analysis of HRQoL, the CHM treatment group exhibited significantly better scores in 4 functional scales (physical, role, emotional, and social), 3 symptom sub-scales (fatigue, pain, nausea and vomiting), the global health condition sub-scale, and 5 individual measurement items (dyspnea, appetite, insomnia, constipation, and economic status) compared to the Non-CHM treatment group (*P* < .05). These findings indicate that CHM treatment can enhance the quality of life in cancer patients, consistent with the results obtained by Wang et al.^[10]^

Both Western medicine and Traditional Chinese Medicine (TCM) possess unique advantages in anti-tumor treatment. Although TCM may not be fully understood by foreign scholars, the efforts of Chinese researchers have provided substantial evidence of its explicit anti-tumor effects in clinical and basic research. TCM, particularly CHM, which includes single herbs such as Panax ginseng (Ren-Shen), Astragalus mongholicus Bunge (Huang-Qi), Angelica sinensis (Oliv.) Diels (Dang-Gui), compound formulations (such as Sijunzi-tang, Bu-zhong-yi-qi-tang, Shi-Quan-Da-Bu-Tang), and Chinese medicine preparations (such as Shenqi Fuzheng Injection, Kanglaite Injection), plays a positive role in regulating the cancer immune system.^[10]^ Furthermore, CHM exhibits its anti-cancer effects through apoptosis induction, proliferation inhibition, metastasis suppression, and multidrug resistance reversal.^[26]^ Clinical studies have shown that CHM treatment improves the quality of life and enhances survival rates in cancer patients.^[27,28]^ Additionally, apart from its anti-tumor properties, CHM has also demonstrated the ability to alleviate depression. For instance, a CHM compound formulation (Xiaoyao Kangai Jieyu Fang) effectively alleviated depression-like behaviors and tumor proliferation in mice.^[29]^ CHM exerts its anti-depression effects by increasing synaptic concentrations of monoamines, alleviating hypothalamic-pituitary-adrenal axis dysfunctions, mitigating neuroplasticity impairment, and modulating immune and inflammatory dysregulation.^[11]^

In our study, we observed that cancer patients diagnosed with stage III and IV were at a higher risk of depression compared to stage I patients. These findings are consistent with those of Tsaras et al^[19]^ The higher risk of depression in stages III and IV can be attributed to a greater symptom burden and poorer physical functioning.^[19]^ In our study, CHM treatment was able to alleviate related symptoms such as fatigue, pain, nausea and vomiting, dyspnea, appetite, insomnia, and constipation. In conjunction with relevant statistics, CHM treatment improved the quality of life and alleviated depression in patients with cancer stages III and IV. Based on clinical experience and the results of univariate analysis regarding depression, metastasis and gene mutation emerged as factors influencing depression. Metastatic cancer or advanced malignancies are particularly susceptible to distress, contributing to the higher prevalence of depression. Our study yielded similar results. Metastasis is strongly associated with inflammation, and inflammation can promote depression in cancer patients.^[4,30]^ CHM exhibits clear anti-inflammatory effects, which may explain its ability to alleviate depression in cancer patients.^[11]^ Previous studies have shown that cancer patients with genetic mutations are at a higher risk of depression, a trend that we also observed in our study.^[31]^ Gene-intracellular environment interactions are a result of tumor development, and CHM treatment can influence tumor cells through modulation of the intracellular environment.^[32,33]^ Considering the relevant statistics, CHM treatment emerges as an independent protective factor against depression in cancer patients. CHM treatment may exert its anti-tumor effects by reducing inflammation, improving the intracellular environment, alleviating related symptoms, and achieving antidepressant effects. However, further research is required to verify these conjectures and gain a deeper understanding of how depression manifests in cancer patients and how CHM impacts their depression status.

Currently, there are numerous cross-sectional studies investigating the depression status in cancer patients in China. However, there is limited research on the relationship between CHM treatment and depression status in these patients. Our study represents the first attempt to explore this association comprehensively in cancer patients. Nevertheless, it is important to acknowledge the limitations of our study. Firstly, being an analytical cross-sectional study, it provides only weak evidence of the relationship between exposure and outcome. Secondly, the recruitment of patients from a single hospital introduces a potential selection bias. Lastly, the sample size in our study might be relatively small, requiring cautious interpretation of our results. Nevertheless, the interesting findings of our study can generate hypotheses for future investigations. Further research, including larger sample sizes and exploring CHM treatment in other race/ethnic groups, is necessary to confirm our findings.

## 5. Conclusion

In conclusion, the depression status of cancer patients significantly impacts their quality of life, treatment compliance, and disease progression. We should pay attention for cancer-related depression in cancer patient. CHM treatment could improve quality of life and aand alleviate depression, while also reducing the side effects of conventional cancer treatments and enhancing immune system function. In the absence of a clear understanding of the impact of antidepressants on tumors, CHM provides a promising alternative approach.

## Acknowledgments

We would like to thank all students (namely Jianying Ma, Jiaying Xu) that helped us recruit patients at Longhua Hospital Shanghai University of Traditional Chinese Medicine (Shanghai, China). We would like to thank all the patients who participated in this study for their support and understanding of our work. We acknowledge the Shanghai Municipal Health Commission and Shanghai Municipal Administrator of Traditional Chinese Medicine, Shanghai, China for supporting our study (no. shslczdzk03701).

## Author contributions

**Conceptualization:** Huiyue Lin, Juyong Wang.

**Data curation:** Huiyue Lin, Xueting Zhang, Yi Zhang, Wenjing Cui.

**Formal analysis:** Huiyue Lin.

**Investigation:** Xueting Zhang, Yi Zhang, Wenjing Cui.

**Methodology:** Xueting Zhang, Yi Zhang, Wenjing Cui, Fang Jia.

**Project administration:** Juyong Wang.

**Supervision:** Fang Jia, Juyong Wang.

**Writing – original draft:** Huiyue Lin.

**Writing – review & editing:** Fang Jia, Juyong Wang.

## References

[1] World Health Organization. Global cancer observatory. Available at: https://gco.iarc.fr/today/home [access date September 15, 2022].

[2] Persky VW Kempthorne-Rawson J Shekelle RB. Personality and risk of cancer: 20-year follow-up of the Western electric study. Psychosom Med. 1987;49:435–49. 3671633 10.1097/00006842-198709000-00001

[3] Helgeson VS Cohen S Schulz R. Group support interventions for women with breast cancer: who benefits from what. Health Psychol. 2000;19:107–14. 10762094 10.1037//0278-6133.19.2.107

[4] Sotelo JL Musselman D Nemeroff C. The biology of depression in cancer and the relationship between depression and cancer progression. Int Rev Psychiatry. 2014;26:16–30. 24716498 10.3109/09540261.2013.875891

[5] Tao WW Jiang H Tao XM. Effects of Acupuncture, Tuina, Tai Chi, Qigong, and traditional Chinese medicine five-element music therapy on symptom management and quality of life for cancer patients: a meta-analysis. J Pain Symptom Manage. 2016;51:728–47. 26880252 10.1016/j.jpainsymman.2015.11.027

[6] Ostuzzi G Matcham F Dauchy S. Antidepressants for the treatment of depression in people with cancer. Cochrane Database Syst Rev. 2015;2015:CD011006. 26029972 10.1002/14651858.CD011006.pub2 PMC6457578

[7] Ng CG Boks MP Zainal NZ. The prevalence and pharmacotherapy of depression in cancer patients. J Affect Disord. 2011;131:1–7. 20732716 10.1016/j.jad.2010.07.034

[8] Luo Y Wang CZ Hesse-Fong J. Application of Chinese medicine in acute and critical medical conditions. Am J Chin Med. 2019;47:1223–35. 31505937 10.1142/S0192415X19500629

[9] Xiang Y Guo Z Zhu P. Traditional Chinese medicine as a cancer treatment: modern perspectives of ancient but advanced science. Cancer Med. 2019;8:1958–75. 30945475 10.1002/cam4.2108 PMC6536969

[10] Wang S Long S Deng Z. Positive role of Chinese herbal medicine in cancer immune regulation. Am J Chin Med. 2020;48:1577–92. 33202152 10.1142/S0192415X20500780

[11] Wang YS Shen CY Jiang JG. Antidepressant active ingredients from herbs and nutraceuticals used in TCM: pharmacological mechanisms and prospects for drug discovery. Pharmacol Res. 2019;150:104520. 31706012 10.1016/j.phrs.2019.104520

[12] Hamilton M. A rating scale for depression. J Neurol Neurosurg Psychiatry. 1960;23:56–62. 14399272 10.1136/jnnp.23.1.56 PMC495331

[13] Aaronson NK Ahmedzai S Bergman B. The European organization for research and treatment of cancer QLQ-C30: a quality-of-life instrument for use in international clinical trials in oncology. J Natl Cancer Inst. 1993;85:365–76. 8433390 10.1093/jnci/85.5.365

[14] Zhao H Kanda K. Testing psychometric properties of the standard Chinese version of the European Organization for Research and Treatment of Cancer Quality of Life Core Questionnaire 30 (EORTC QLQ-C30). J Epidemiol. 2004;14:193–203. 15617393 10.2188/jea.14.193 PMC8784239

[15] Eberst G Anota A Scherpereel A.; French Cooperative Thoracic Intergroup (IFCT). Health-related quality of life impact from adding bevacizumab to cisplatin-pemetrexed in malignant pleural mesothelioma in the MAPS IFCT-GFPC-0701 phase III trial. Clin Cancer Res. 2019;25:5759–65. 31175096 10.1158/1078-0432.CCR-18-2860

[16] Kernan WN Viscoli CM Brass LM. Phenylpropanolamine and the risk of hemorrhagic stroke. N Engl J Med. 2000;343:1826–32. 11117973 10.1056/NEJM200012213432501

[17] Faul F Erdfelder E Lang AG. G*Power 3: a flexible statistical power analysis program for the social, behavioral, and biomedical sciences. Behav Res Methods. 2007;39:175–91. 17695343 10.3758/bf03193146

[18] Linden W Vodermaier A Mackenzie R. Anxiety and depression after cancer diagnosis: prevalence rates by cancer type, gender, and age. J Affect Disord. 2012;141:343–51. 22727334 10.1016/j.jad.2012.03.025

[19] Tsaras K Papathanasiou IV Mitsi D. Assessment of depression and anxiety in breast cancer patients: prevalence and associated factors. Asian Pac J Cancer Prev. 2018;19:1661–9. 29938451 10.22034/APJCP.2018.19.6.1661 PMC6103579

[20] Korsten LHA Jansen F de Haan BJF. Factors associated with depression over time in head and neck cancer patients: a systematic review. Psychooncology. 2019;28:1159–83. 30865357 10.1002/pon.5058 PMC6593868

[21] Götze H Friedrich M Taubenheim S. Depression and anxiety in long-term survivors 5 and 10 years after cancer diagnosis. Support Care Cancer. 2020;28:211–20. 31001695 10.1007/s00520-019-04805-1

[22] Yang SY Livneh H Jhang JS. Association of Chinese herbal medicine use with the depression risk among the long-term breast cancer survivors: a longitudinal follow-up study. Front Psychol. 2022;13:884337. 36059752 10.3389/fpsyg.2022.884337 PMC9434377

[23] Tao W Luo X Cui B. Practice of traditional Chinese medicine for psycho-behavioral intervention improves quality of life in cancer patients: a systematic review and meta-analysis. Oncotarget. 2015;6:39725–39. 26498685 10.18632/oncotarget.5388 PMC4741858

[24] Zhang S Chen H Zhang M. Reduction of depression symptoms in laryngeal cancer patients receiving psychology services. Am J Transl Res. 2020;12:6637–45. 33194060 PMC7653580

[25] Li M Kouzmina E McCusker M. Cytokines and depression in cancer patients and caregivers. Neuropsychiatr Dis Treat. 2017;13:2903–11. 29238195 10.2147/NDT.S144774 PMC5713706

[26] Yan Z Lai Z Lin J. Anticancer properties of traditional Chinese medicine. Comb Chem High Throughput Screen. 2017;20:423–9. 28093974 10.2174/1386207320666170116141818

[27] Jiang Y Liu LS Shen LP. Traditional Chinese medicine treatment as maintenance therapy in advanced non-small-cell lung cancer: a randomized controlled trial. Complement Ther Med. 2016;24:55–62. 26860802 10.1016/j.ctim.2015.12.006

[28] Liu CT Chen YH Huang YC. Chemotherapy in conjunction with traditional Chinese medicine for survival of patients with early female breast cancer: protocol for a non-randomized, single center prospective cohort study. Trials. 2019;20:741. 31847861 10.1186/s13063-019-3848-8 PMC6918648

[29] Meng P Han Y Yang Q. Xiaoyao Kangai Jieyu Fang, a Chinese herbal formulation, ameliorates cancer-related depression concurrent with breast cancer in mice via promoting hippocampal synaptic plasticity. Evid Based Complement Alternat Med. 2018;2018:3967642. 30581482 10.1155/2018/3967642 PMC6276466

[30] McFarland DC Saracino RM Miller AH. Prognostic implications of depression and inflammation in patients with metastatic lung cancer. Future Oncol. 2021;17:183–96. 33305608 10.2217/fon-2020-0632 PMC7857340

[31] Zhou Y Gu X Wen F. Association of KRAS gene mutations with depression in older metastatic colorectal cancer patients. Int Psychogeriatr. 2016;28:2019–28. 27468967 10.1017/S1041610216001125

[32] Masclef L Ahmed O Estavoyer B. Roles and mechanisms of BAP1 deubiquitinase in tumor suppression. Cell Death Differ. 2021;28:606–25. 33462414 10.1038/s41418-020-00709-4 PMC7862696

[33] Xie H Feng S Farag MA. Synergistic cytotoxicity of erianin, a bisbenzyl in the dietetic Chinese herb Dendrobium against breast cancer cells. Food Chem Toxicol. 2021;149:111960. 33385512 10.1016/j.fct.2020.111960

